# Ultrasound-based Node-RADS: Introducing a new Scoring System for Ultrasound-based Classification of Lymphadenopathy

**DOI:** 10.22038/ijorl.2025.85674.3883

**Published:** 2025

**Authors:** Amir Mohammad Heravi, Fatemeh Hataminia, Bashir Rasoulian, Maryam Tavakoli, Narjes Sadat Yaghobi, Amir Hossein Jafarian, Seyed Ali Alamdaran

**Affiliations:** 1 *Department of Radiology, Mashhad University of Medical Sciences, Mashhad, Iran.*; 2 *Faculty of Medicine, Mashhad University of Medical Sciences, Mashhad, Iran.*; 3 *Department of ENT, Mashhad University of Medical Sciences, Mashhad, Iran.*; 4 *Department of Pathology, Mashhad University of Medical Sciences, Mashhad, Iran.*

**Keywords:** Lymphadenopathy classification, Ultrasound, Node- Reporting and Data System (Node-RADS)

## Abstract

**Introduction:**

Lymphadenopathy often causes anxiety due to its association with malignancy or serious infections. This study investigates the role of ultrasound features in distinguishing benign from malignant neck lymphadenopathy and proposes a quantitative scoring system (Node-RADS).

**Materials and Methods:**

This cross-sectional study was conducted at Omid Hospital, Mashhad University of Medical Sciences, Iran. Seven hundred ninety-one patients with neck lymphadenopathy underwent gray-scale and Doppler ultrasound, followed by fine needle aspiration (FNA) or core needle biopsy (CNB) for cytopathological confirmation. Key ultrasound features assessed included Short-Axis Diameter (SAD), Cortical/Hilar Echotexture, and Vascular patterns. A scoring system was developed by assigning malignancy coefficients to each variable. Malignancy coefficients (Wi) were assigned based on the prevalence of malignancy for each feature, and a quantitative Node-RADS score was derived. Diagnostic accuracy was evaluated using ROC analysis.

**Results:**

Of 791 patients, 68.5% (542) had malignant lymphadenopathy, predominantly metastases (57.1%, 452). Malignancy coefficients (Wi = 9) were extracted to high-risk features: SAD >16 mm (82% malignancy), Isoechoic cortex with compressed hilum (89%), and non-hilar vascularity (91%). The proposed Node-RADS system achieved an AUC of 0.85 (95% CI: 0.817–0.889), demonstrating strong diagnostic performance.

**Conclusion:**

The proposed ultrasound-based Node-RADS scoring system correlates significantly with pathologic results, offering an appropriate tool for evaluating cervical superficial lymphadenopathy.

## Introduction

Lymphadenopathy is a common clinical finding. Its importance is due to its association with malignancy or severe infections. While most cases are benign, accurate differentiation is critical for cancer staging (TNM; Tumor, Node, Metastasis) and management. 

Clinicians must carefully determine whether immediate invasive intervention is necessary or whether clinical observation and follow-up are sufficient (1,2).

In addition to history-taking and physical examination, patients typically undergo complementary diagnostic evaluations, including laboratory tests and imaging studies such as ultrasound, chest X-ray, CT scans, Fine Needle Aspiration, and Core Needle Biopsy (3–5). While a definitive diagnosis requires histopathological examination, invasive procedures such as biopsies can place significant physical and emotional burdens on patients. Imaging modalities, particularly ultrasound, play a key role in non-invasive evaluation due to their accessibility and high resolution in superficial regions like the neck (3-7). 

In recent years, in many organs, lesions have been classified based on the risk of malignancy using standardized classification systems such as the ACR-RADS series. These systems help improve diagnostic accuracy, guide treatment decisions, facilitate better physician communication, prevent overtreatment, and support more rigorous scientific research (8). 

Given the high prevalence and varied causes of lymphadenopathy, a standardized classification system - such as the Node-Reporting and Data System (Node-RADS) - would be beneficial. However, most existing Node-RADS classification is based on CT and MRI findings, using "size" and "configuration" as key criteria to classify nodes from 1 ("very low probability") to 5 ("very high probability"). 

Similar to other classifications, these systems categorize lymph nodes based on their likelihood of malignancy (9-17). However, existing research on ultrasound-based Node-RADS has limited support and requires further research, particularly in ultrasound-based applications. Since the neck is a common site for both malignant and inflammatory lymphadenopathy and the use of high-resolution ultrasound (>10 MHz) in this area, a standardized ultrasound-based Node-RADS system could be especially valuable. To date, only three ultrasound-based lymph node classification systems have been reported in the literature: the Cervical Lymph Node Imaging Reporting and Data System (CLN-RADS), the Adenopathy Reporting and Data System (A-RADS), and the Lymph Node Reporting and Data System (LN-RADS) (2,18,19). CLN-RADS is considered outdated, and A-RADS is primarily qualitative. The A-RADS study showed that features such as increased short axis, absence of a hilum with isoechoic cortex, and presence of non-hilar vessels were significantly more common in malignant lymph nodes than benign ones. This study classified cervical lymph nodes into four groups: normal, reactive, suspicious/lymphatic abnormalities, and metastatic (19). 

This study aimed to evaluate the diagnostic value of specific ultrasound features in differentiating types of cervical lymphadenopathy and propose a quantitative, ultrasound-based scoring system. 

## Materials and Methods

This study investigated neck lymphadenopathy in patients referred to the radiology Clinic at Omid Hospital, Mashhad University of Medical Sciences. The institution’s Ethics Committee approved the study protocol (IR. MUMS.IRH. REC.1403.070). Informed consent was obtained from all participants. All patient information was anonymized using coded identifiers for data analysis, and results were reported in aggregate to preserve confidentiality. Initially, demographic information of 791 patients, such as age and gender, was recorded. Participants underwent gray-scale and Doppler ultrasound imaging using an Esaote Class C ultrasound system with a linear probe (8–16 MHz). 

An experienced radiologist performed ultrasound examinations. Diagnostic approaches included Clinical and Laboratory Work-up, Repeated Ultrasound, Tg washout, Fine Needle Aspiration (FNA), Core Needle Biopsy (CNB), and Therapeutic Neck Dissection. Based on these results, patients were categorized into two groups: benign (reactive, inflammatory, lymphadenitis, granulomatosis) and malignant (metastasis, lymphoma). Subsequently, ultrasound findings were compared between these two groups. An echogenic hilum, a symmetrical hypoechoic cortex, and minimal hilar vascularity characterize Normal lymph nodes. Echotextures were classified into the hypoechoic cortex, invisible hilum, and Hyperechoic cortex. Normal cervical lymph nodes in the jugulodigastric region usually have a short axis diameter (SAD) of approximately 10 mm, whereas lymph nodes in other neck areas are generally smaller (<8 mm). Accordingly, cutoff values of 10 mm (jugulodigastric region) and 8 mm (other regions) were used. Vascular patterns on Doppler were categorized as hilar, non-hilar, or decreased flow. To develop a quantitative Node-RADS classification system, each lymph node ultrasound feature was assigned a malignancy risk coefficient (Wi) based on its prevalence (Pi) in malignant cases. Total malignancy scores ($S_{j}$) were calculated using the following formula: 

$S_{j}=(\sum_{i=1}^{\mathrm{Nj}}X_{j}{N_{\mathrm{ij}}W}_{i})$

Here, *X*_j_ indicates the feature’s presence (1) or absence (0), $N_{\mathrm{ij}}$represents the feature count, and $W_{i}$is the malignancy coefficient. The malignancy coefficient was assigned based on Pi as follows. Pi for each feature: Pi ≤ 30%: $W_{i}$= 1 , 30% < Pi < 50%: $W_{i}$= 3 , 50% ≤ Pi < 70%: $W_{i}$= 5 , 70% ≤ Pi < 80%: $W_{i}$= 7 , 80% ≤ Pi < 100%: $W_{i}$= 9 . 

These scores were simplified to facilitate application and classification into Node-RADS categories (I–V) (1,3,5,7,9→0,1,2,3,4). 

Because normal lymph nodes are typically hypoechoic and have a short axis diameter of less than 8 or 10 mm, and since the study's data was primarily derived from lymphadenopathy cases, the scores for these two features were considered lower than their calculated coefficients to help differentiate normal lymph nodes from abnormal ones within the Node-RADS classification. The final Node-RADS score was determined by comparing the summed malignancy scores between benign and malignant groups. Receiver Operating Characteristic (ROC) curves were used to assess the predictive accuracy of the proposed classification system. 

## Results

This prospective study included 791 patients with neck adenopathy (45.9 ± 18.1 years; 40.2% -318 male, 59.8% - 473 female). Malignant tumors constituted the majority (542 cases, 68.5%), while 249 cases (31.5%) were benign. Key diagnoses include metastasis, the most common diagnosis, accounting for 452 cases (57.1% of the total). Reactive conditions were the second most frequent, with 187 cases (23.6%). Hodgkin's Lymphoma (HL) and Non-Hodgkin's Lymphoma (NHL) represented 40 cases (5.0%) and 50 cases (6.4%), respectively. Other diagnoses included Granulomatosis (41 cases, 5.2%), Thyroid tissue (7 cases, 0.9%), Salivary Gland pathology (6 cases, 0.8%), and Seroma (5 cases, 0.6%). Rare diagnoses such as Bronchial Cyst and Plasmacytoma were each found in only one case (0.1%).

Table 1 provides an analysis of malignancy rates in neck lymph nodes, derived parameters derived from pathological findings and ultrasound (US) features. Key metrics include malignancy incidence (Pi), which reflects the percentage of patients with malignancy, and malignancy coefficients (Wi), which measure the association of specific features with malignancy risk. Below is a breakdown of the findings: 

*Short Axis Diameter (SAD):* Smaller nodes (SAD < 8 or 10 mm) had a malignancy incidence of 63-65% and a coefficient of 5. Bigger nodes (SAD ≥ 8 or 10 mm) showed a higher malignancy incidence of 70-71% and a coefficient of 7. A high malignancy condition was found in nodes with SAD >16 mm, with a coefficient of 9. 

*Echo-texture:* Hypo-echoic cortex showed a malignancy incidence of 54% with a coefficient of 5. Invisible hilum with Iso-echoic cortex had a high malignancy incidence of 89% and a coefficient of 9. 

*Vascularity Patterns:* Nodes with hilar vascularity showed a malignancy incidence of 20% and the lowest coefficient (Wi = 1). Nodes with non-hilar vascularity had the highest malignancy incidence of 91% and a coefficient of 9. Non/Reduced vascularity nodes showed an incidence of 64% with a coefficient of 5.

**Table 1:** Malignancy Analysis by Ultrasound and Pathological Features of Neck Lymph Nodes: Key parameters included malignancy incidence (Pᵢ) and malignancy coefficients (Wᵢ) derived from ultrasound (US) and pathological findings

**Lymph node Type**	**Lymph node Features (i=12)**	**Number of Patients**	**Patients with Malignancy Lymph**	**Incidence of Malignancy (Pi)**	**Malignancy Coefficient (Wi)**	**Proposed Score**
SAD	W < 10) (Jugulodigastric area)	303	196	64.6/%	5	0
	W < 8 (Others area)	190	120	63%	5	0
	W ≤ 10 (Jugulodigastric area)	480	341	71.0%	7	2
	W ≤ 8 (Others area)	593	417	70.0 %	7	2
	W ≤ 16	228	187	82%	9	4
Echo-texture	Hypoechoic cortex	451	244	54%	5	1
	Iso-echoic cortex	307	272	89%	9	4
Vascularity	Hilar	139	28	20%	1	0
	Non-Hilar	339	310	91%	9	4
	Non/Reduced Flow	154	99	64.0%	5	1

The relationship between malignancy coefficients (Sj) and the Node-RADS classifications based on ultrasonography findings was also calculated. Each category assesses malignancy risk, with Nj representing the number of patients and Sj indicating the mean malignancy coefficient with its standard deviation (SD). The Node-RADS classification is as follows: Node-RADS 1: Sj ≤ 11 (mean ± SD), suggesting no malignancy risk. Node-RADS 2: Sj 11-16 (15.7 ± 5.3), indicating a very low malignancy risk with some variability. Node-RADS 3: Sj 16-20 (20.1 ± 4.5), showing a low malignancy risk with slightly higher variability. Node-RADS 4: Sj 20-25 (24.5 ± 5.3), with moderate malignancy risk and variability comparable to Node-RADS 2 and 3. Node-RADS 5: Sj ≥ 25, indicating the highest malignancy risk.

**Fig 1:**
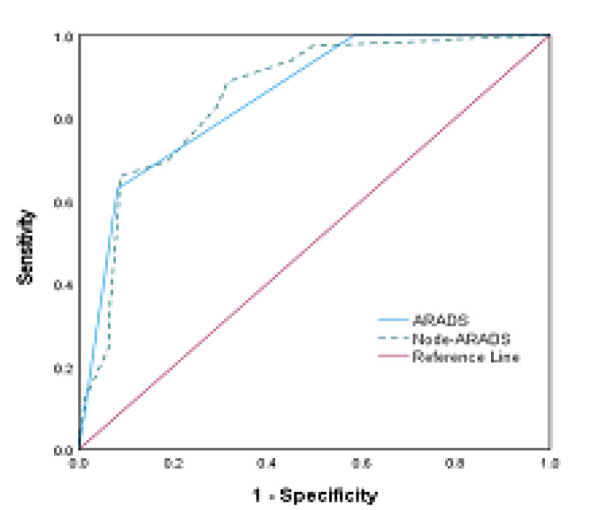
ROC analysis of A-RADS and Node-RADS as test result variables compared to final diagnosis results. The Node-RADS method achieves superior diagnostic performance compared to the A-RADS method, as its curve is closest to the top-left corner of the plot.

The Qualitative (A-RADS) and Quantitative (Node-RADS) diagnostic accuracy was compared. Figure 1 illustrates the ROC (Receiver Operating Characteristic) curves for diagnostic methods, comparing A-RADS (Qualitative) and Node-RADS (Quantitative) with final diagnosis results for assessing malignancy. The Node-RADS method achieved a higher AUC of 0.853 (95% CI: 0.817–0.889), indicating superior diagnostic accuracy. The A-RADS method had an AUC of 0.851 (95% CI: 0.816–0.885), demonstrating strong diagnostic performance. Both methods demonstrated excellent diagnostic performance (p<0.001), with Node-RADS showing marginally superior accuracy. The ROC curve for Node-RADS (dashed line) lies closer to the top-left corner, reflecting its superior predictive capability (Figure 1).

## Discussion

Although lymphadenopathy is a key component of the TNM system in cancer staging and management, it is also a common self-limiting finding in infections. Its diverse etiologies necessitate careful judgment to determine whether immediate intervention or observation is appropriate (1,2). Definitive diagnosis and differentiation between benign and malignant lymphadenopathy rely on histopathological examination, though biopsies impose patient burdens (6,7). 

Classification systems such as the Node Reporting and Data System (Node-RADS) offer structured frameworks to guide patient management (8). 

These systems are crucial in clinical settings to reduce unnecessary invasive procedures (e.g., biopsies), improve patient management and treatment planning, and decrease healthcare costs through accurate lymph node evaluation. 

Limited research exists on Node-RADS, which primarily utilizes CT and MRI findings. Elsholtz et al. (9) proposed a Node-RADS scoring system based on lymph node "size" and "configuration" on CT and MRI images to stratify malignancy risk on a scale from 1 ("very low probability") to 5 ("very high probability"). Niu et al. (10) compared the diagnostic performance of the Node-RADS scoring system with lymph node (LN) size in the preoperative assessment of 146 patients with rectal cancer. In their study, the value of size-based assessment was more accurate than morphology-based assessment. Wu and colleagues (11) analyzed MRI scans of 81 patients with cervical cancer and confirmed categories 4 and 5 of the Node-RADS classification as effective predictors of lymph node metastasis (LNM). Loch et al. studied 91 patients with gastric adenocarcinoma and found that short-axis diameter (≥10 mm) achieved 57% sensitivity and 87% specificity for malignancy (12). 

Chung et al. analyzed CT images of 476 cervical lymph nodes from 191 patients with head and neck squamous cell carcinoma. The criteria included shortest axial diameter, the ratio of long-to-short axis diameter, necrosis, conglomeration, infiltration into adjacent soft tissue, laterality, and T-stage of the primary tumor. The risk of malignancy ranged from 7.3% to 99.8%, positively correlated with higher scores (13). Zhong et al. (14) conducted a meta-analysis of Node-RADS (6 CT, 3 MRI studies), reporting malignancy rates of 4% (Node-RADS 1) to 100% (Node-RADS 5), though inter-observer reliability remained suboptimal. 

Yang et al. evaluated 203 cervical lymph nodes from 119 nasopharyngeal cancer patients using Node-RADS on MRI. A Node-RADS cutoff of 3 yielded a sensitivity of 92% and specificity of 87% for metastasis (15). Yu et al. analyzed 1033 cervical lymph nodes from CT images of 348 papillary thyroid carcinoma patients using a deep learning-based automatic system. They reported a sensitivity and specificity of 0.68 and 0.48, respectively (16).

Parillo et al. reviewed 17 relevant articles and concluded that Node-RADS shows promising diagnostic performance for both Node-RADS ≥ 3 and Node-RADS ≥ 4 as positive thresholds, but the optimal cutoff value for determining lymph node metastasis remains uncertain (17).

The Neck Imaging and Reporting and Data System (NI-RADS), introduced in 2018 by the American College of Radiology, is a risk score system to standardize reporting of post-treatment contrast-enhanced CT or MRI with or without PET in head and neck cancer patients. Categories 1–4 guide management based on recurrence risk: Category 1: Expected post-treatment changes; routine follow-up. 

Category 2: Moderate FDG-PET uptake; endoscopic evaluation or short-term follow-up. Category 3: New/enlarging mass or intense PET uptake; biopsy recommended. Category 4: Definitive progression; new treatment plan required. Ultrasonography, a non-radiation modality, excels in assessing superficial lymph nodes (e.g., neck), evaluating their size, shape, margins, echogenicity, and vascularity. 

The neck, which contains approximately 300 of the body's 800 lymph nodes, is a common site of malignant and inflammatory adenopathy, making ultrasound particularly valuable (19-21). 

Few studies have explored ultrasound-based Node-RADS; Ryu et al. proposed CLN-RADS for cervical lymphadenopathy using Ultrasound and Elastography. They studied 291 consecutive patients who underwent US-guided biopsies for cervical lymphadenopathy. Malignancy risk scored with the following imaging features: shape (round = 1, oval = 0), echogenicity (hyper-echogenicity = 1, iso-echogenicity or hypo-echogenicity = 0), the presence or absence of an echogenic hilum (absence = 1, presence = 0), presence of calcification (presence = 1, absence = 0), and peripheral and mixed vascular patterns (presence = 1, absence = 0); and the elasticity scores and strain ratio (RTE) (malignant RTE assessment = 1, benign RTE assessment = 0). The risk of malignancy increased as the number of suspicious features increased. According to the Cervical Lymph Node Imaging Reporting and Data System (CLN-RADS), malignancy risk ranged as follows: category 1, 3.3% (probably benign / no suspicious feature); category 2, 10.9% (low suspicion / one suspicious feature); category 3, 26.7% (moderate suspicion / two suspicious features); category 4, 51.8–74.4% (high suspicion / three or four suspicious features); and category 5, 90.6–98.8% (very high suspicion / five or six suspicious features) (18). In this study, the clinical application of this classification in the management of patients was not stated (18). 

Adenopathy Reporting and Data System (A-RADS), A qualitative classification, categorized nodes based on gray-scale and Doppler ultrasound appearances: I (Normal): SAD < 1 cm with few hilar vessels. II (Reactive): SAD = 1-1.5 cm, hypoechoic cortex with visible hilum and increased hilar vessels. III (Suspicious & Lymphoid Disorders): SAD > 1 cm, hypoechoic cortex with small or invisible hilum. IV (Metastatic): Iso-echoic cortex and invisible hilum with non-hilar vessels (9). 

Chudobiński et al. compared Artificial Intelligence (AI) findings of ultrasound images and size in superficial adenopathies. They demonstrated that AI findings were more valuable than the size threshold (10 mm) in differentiating superficial adenopathies. 

This study proposes a quantitative scoring system for node-RADS classification, where malignancy coefficients based on the prevalence of malignancy were assigned to specific variables. As shown in Table 1, malignancy coefficients for ultrasound features of lymph nodes include Short-axis diameter (SAD): >16 mm (82% malignancy; coefficient 9). Echo-texture (invisible hilum, iso-echoic cortex): (89% malignancy; coefficient = 9). Vascularity (non-hilar flow): (91% malignancy; coefficient 9); reduced vascularity (67%; coefficient 5). 

These malignancy coefficients were converted into scores (1, 3, 5, 7, 9 to 0, 1, 2, 3, 4), resulting in the following scores: SAD > 16 mm = 4, hyperechoic cortex = 3, non-hilar vascularity = 4. Normal hypoechoic lymph nodes are generally less than 8 or 10 mm in short axis and have few vessels. Based on this data, it is recommended that the hypoechoic cortex, SAD < 8–10 mm, and decreased vascularity receive lower malignancy coefficients to distinguish between normal lymph nodes and lymphadenopathy. As the Node-RADS category increases from 1 to 5, both the malignancy coefficients (Sj) for malignancy show a consistent upward trend, indicating a correlation between higher Node-RADS classifications and increased malignancy likelihood. Node-ARADS 1 shows no malignancy (Sj > 11), while Node-ARADS 5 presents the highest malignancy coefficients (Sj < 25). In this study, scores (0–4) derived from coefficients yielded an AUC of 0.873 (95% CI: 0.840–0.905), aligning with Ryu et al.'s study (18). Cystic changes/calcifications often correlate with malignancy (18,22–25). Since most patients with these two features have a hyperechoic cortex, their significance in hypoechoic nodes requires further study. In addition, in some studies, the presence or absence of calcification or cystic change in malignant and non-malignant adenopathies did not differ significantly (19). 

Both qualitative and quantitative classifications showed excellent diagnostic performance (Node-RADS AUC = 0.853 vs. A-RADS AUC = 0.851), and overlapping 95% confidence intervals indicated no significant difference (p < 0.001). Although the differences between the methods are small, the quantitative system demonstrates incremental improvement in malignancy prediction, supporting the utility of the Node-RADS approach. 

Table 2 outlines the proposed ultrasound-based Node-RADS scoring system. While fine needle aspiration (FNA) aids malignancy detection, core needle biopsy (CNB) remains essential for persistent nodes (>2–4 weeks) and lymphoid disorders (20). Although fine needle aspiration (FNA) and cytology are valuable in malignancy detection, they have limitations in differentiating the type of malignancy, particularly in lymphatic disorders. Therefore, Core needle Biopsy (CNB) and histopathology remain essential for adenopathies of unknown origin and lymphoid disorders. 

In several studies, persistent lymph nodes (>2–4 weeks) are an important clinical indicator of malignancy and often necessitates biopsy (20). 

This study lays the framework for future research to confirm the clinical effectiveness of this scoring system across different populations and other superficial areas. However, the ultrasound-based Node-RADS scoring system is a new diagnostic approach, and further research is needed to confirm its clinical efficacy. 

**Table 2:** Proposed Classification System for Ultrasound-based Node-RADS.

**Node RADS Classification of lymphadenopathy & Recommendation**					
**Ultrasound Features**	**Score**	**Total Score**	**Node-RADS**	**Recommendation**	
**SAD (Short Axis Diameter)**		≤1	I Normal	None	
Jugulodigastric < 10 mm Other < 8 mm	0				
Jugulodigastric ≥ 10 mm Other ≥ 8 mm	2	2	II Reactive	Clinical follow-up ± Treatment	
≥ 16 mm	4				
**Echo-texture**		3	III Probably Reactive	Repeated Ultrasound in 2-4 weeks	
Hypo-echoic cortex	0				
Invisible hilum	1				
Hyper-echoic cortex	3	4	IV Suspicious	Known Origin	FNA
**Vascularity**					
Hilar	0			Unknown Origin	CNB
Decreased	1				
Non-hilar	4	≥5	V Metastatic / Tumoral	Known Origin	Treatment
**Clinical History **					
				Unknown Origin	CNB
< 2-4 week	0				
				Post -Op (Known Origin)	FNA
> 2-4 week	1				

## Conclusion

 The proposed ultrasound-based Node-RADS scoring system correlates significantly with pathologic results, offering an appropriate tool for evaluating cervical lymphadenopathy. 
